# The Impact of Intracranial Pressure Telesensors: An Observational Propensity-Matched Control Analysis of Service Demand and Costs

**DOI:** 10.1227/neu.0000000000002893

**Published:** 2024-03-06

**Authors:** Anand S. Pandit, Muhammad A. Kamal, Gerda Reischer, Yousif Aldabbagh, Mohammad Alradhawi, Faith M. Y. Lee, Priya P. Sekhon, Eleanor M. Moncur, Ptolemy D. W. Banks, Simon Thompson, Lewis Thorne, Laurence D. Watkins, Ahmed K. Toma

**Affiliations:** * Victor Horsley Department of Neurosurgery, National Hospital for Neurology & Neurosurgery, London, UK;; ‡ High-Dimensional Neurology, Institute of Neurology, University College London, London, UK;; § UCL Medical School, Faculty of Medical Sciences, University College London, London, UK

**Keywords:** Adult hydrocephalus, CSF disorders, Shunt, Cost-effectiveness

## Abstract

**BACKGROUND AND OBJECTIVES:**

Implantable telemetric intracranial pressure (ICP) sensors (telesensors) enable routine, noninvasive ICP feedback, aiding clinical decision-making and attribution of pressure-related symptoms in patients with cerebrospinal fluid shunt systems. Here, we aim to explore the impact of these devices on service demand and costs in patients with adult hydrocephalus.

**METHODS:**

We performed an observational propensity-matched control study, comparing patients who had an MScio/Sensor Reservoir (Christoph Miethke, GmbH & Co) against those with a nontelemetric reservoir inserted between March 2016 and March 2018. Patients were matched on demographics, diagnosis, shunt-type, and revision status. Service usage was recorded with frequencies of neurosurgical admissions, outpatient clinics, scans, and further surgical procedures in the 2 years before and after shunt insertion.

**RESULTS:**

In total, 136 patients, 73 telesensors, and 63 controls were included in this study (48 matched pairs). Telesensor use led to a significant decrease in neurosurgical inpatient admissions, radiographic encounters, and procedures including ICP monitoring. After multivariate adjustment, the mean cumulative saving after 2 years was £5236 ($6338) in telesensor patients (£5498 on matched pair analysis). On break-even analysis, cost-savings were likely to be achieved within 8 months of clinical use, postimplantation. Telesensor patients also experienced a significant reduction in imaging-associated radiation (4 mSv) over 2 years.

**CONCLUSION:**

The findings of this exploratory study reveal that telesensor implantation is associated with reduced service demand and provides net financial savings from an institutional perspective. Moreover, telesensor patients required fewer appointments, invasive procedures, and had less radiation exposure, indicating an improvement in both their experience and safety.

**ABBREVIATIONS:**
ICPM: intracranial pressure monitoring
SpR: specialist registrar
VPS: ventriculo-peritoneal shunt.

The implantation of a shunt system represents the principal treatment option for long-term cerebrospinal fluid (CSF) diversion and regulation of intracranial pressure (ICP) in adult hydrocephalus.^1^ Incorporating a magnetically adjustable valve in series with the shunt allows for fine-tuning of CSF drainage to regulate ICP,^2^ and implantable reservoirs allow for percutaneous CSF sampling if necessary. After shunt insertion, valve adjustments are typically made based on clinical or radiological evidence of CSF under-drainage or over-drainage. However, patient symptom information is often inaccurate, misattributed, and often not well correlated with ICP.^3,4^ Conventional computed tomography (CT)–based imaging can provide evidence of high-pressure or low-pressure states, but it exposes patients to unnecessary ionizing radiation, resource use, and additional outpatient appointments.

Telemetric sensors (“telesensors”) are implantable devices that can measure ICP noninvasively. Although some telesensors such as the Neurovent-P-tel sensor (Raumedic AG) are restricted for short to intermediate periods of use before removal,^5^ others are permanently implanted. One such latter device is the MScio® (Christoph Miethke GmbH): a telesensor reservoir, which replaces a traditional reservoir and is implanted in series as part of a shunt system. The MScio offers routine ICP measurement and has shown to be useful in both adults and children,^6,7^ is accurate against the gold standard of bolt-based ICP measurement,^8^ is MRI compatible,^9^ and can conveniently be used in clinic and in different patient positions.^10^

The advantages of permanent telesensor devices are manifold. Clinically, they provide rapid, noninvasive measurement of ICP, thereby offering an almost instantaneous method to identify and triage patients with abnormal pressure levels. Normal telemetric readings, on the other hand, can reassure both patients and the surgical team that symptoms, if present, are not linked with shunt dysfunction. From a service perspective, the use of permanent telesensor devices like the MScio may reduce neuroimaging, hospital admissions, and further clinic appointments, reducing overall service demand. While these latter benefits have been posited, it remains unclear whether the MScio is truly cost-effective and whether and when the initial up-front cost can be recouped.

Here, we present our institutional experience regarding the use of MScio telesensors for adult hydrocephalus, which, to the best of our knowledge, has the largest volume of telesensor patients worldwide. Through a comprehensive exploratory analysis, we investigate whether telesensor usage is associated with a difference in service and financial demands as compared with patients with nontelemetric reservoirs.

## METHODS

### Guidelines

Where relevant, this retrospective matched control study was conducted in accordance with Recommendations for Reporting Cost-Effectiveness Analyses by the Panel of Cost-Effectiveness in Health and Medicine.^11^

### Ethics

The local institutional review board approved this service evaluation in the use of telesensor devices at our center (122-202021-CA). All patients consented to their surgical intervention.

### Patients

This study was conducted in a large-volume tertiary neurosciences center in London, United Kingdom. Included were all patients who were (1) treated for hydrocephalus using a primary or revision CSF diversion technique (ventriculo-peritoneal shunt, lumbo-peritoneal shunt, endoscopic third ventriculostomy with catheter insertion) that involved use of an MScio sensor reservoir; (2) the telesensor was implanted between March 2016 and 2018; and (3) could be followed up for a minimum of 2 years after implantation. Patients who had an MScio reservoir implanted were chosen during a period of clinical equipoise, rather than based on predetermined guidelines or specific to a certain attending. Control patients were selected based on the aforementioned inclusion criteria but did not have a telemetric reservoir. For the overwhelming majority of controls, a Sprung reservoir (Christoph Miethke GmbH) was implanted.

The 2-year window after implantation was selected to allow an adequate period to test whether differences existed in use of services and their associated costs. This period was also selected to prevent overlap with the COVID-19 pandemic period and associated governmental lockdown restrictions which could bias the number and type of clinical encounters. An equal length window before implantation was selected by the study team to ensure a sufficient period, allowing assessment of the control matching process.

#### Telesensor

**FIGURE 1.**
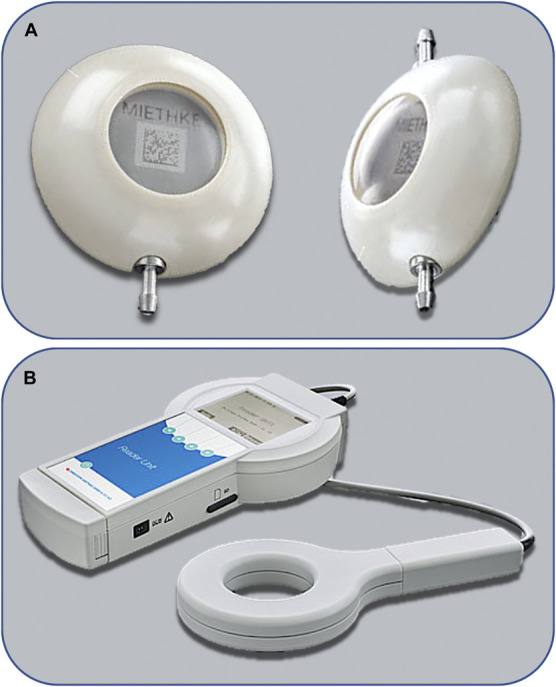
MScio® sensor reservoir (Miethke GmbH) and reader system. **A**, Dome angled MScio implant (left) and in-line MScio implant (right); **B**, MScio Reader Unit set. *Images obtained and adapted with permission from Miethke GmbH.*

The MScio® (Christoph Miethke GmbH, previously “Sensor Reservoir”) is a coin-sized device implanted along the tubing or at the angle of the shunt and the tubing at the burr hole site (Figure 1A). A compressible metal membrane is depressed by adjacent CSF when ICP increases. This mechanical stimulation is sensed by a measuring cell and, when in proximity, communicates the real-time pressure measurement telemetrically to a hand-held receiver (Figure 1B).

### Data Collection

Data were collected independently and double-checked to ensure consistency. Patient demographics and clinical and operative information were retrieved from the institution's electronic health record (Epic System Corporation) along with the dates and numbers of scans, admissions, and encounters in the period before and after telesensor implantation. For neurosurgical encounters, the date, type (admission, outpatient clinic, or correspondence), personnel (attending, resident or nurse specialist), whether the telesensor was read, and any relevant invasive procedures were recorded. For imaging encounters, the date and scan type (MRI, CT, x-ray) were recorded. Encounters relating to the patient's hydrocephalus condition seen by neurology, ophthalmology, and emergency medicine staff were also included. Excluded were same-institution encounters unrelated to hydrocephalus management.

### References and Tariffs

The following radiation reference values were used: a patient undergoing a plain CT head scan would receive 2.0 mSv and for an x-ray shunt series of the skull, chest, and abdomen: 0.9 mSv. For differences in patient costs, calculations were performed based on negotiated local tariffs wherever available for 2021–2022 (Table 1). Cost differences were analyzed at both annual and biannual intervals. United States dollar conversion rates were based on the tariff date of 1/7/22.

**TABLE 1.** Institutional Tariffs for the Telesensor Device and Various Hospital Encounters Inclusive of Staff Costs

Type of encounter	Subtype	Tariff in GBP (US$)	Notes
Hospital attendance	Neurosurgical outpatient clinic	£192 (232.4)	Routine (may include valve adjustment), single professional
	Neurosurgical admission long-stay tariff per day	£648 (784.4)	Tariffed at more than 5 days admission, inclusive of labour and overheads
	Emergency admission per day	£172 (208.2)	Emergency medicine category II investigation with category II treatment
	Neurology outpatient clinic	£189 (228.7)	Routine, single professional
	Ophthalmology outpatient clinic	£139 (168.3)	Routine, single professional
Radiology	MRI scan	£114 (137.9)	Single area without contrast, exclusive of reporting fees
	CT scan	£72 (87.1)	
	Skull/chest/abdominal x-ray	£42 (50.8)	—
Invasive	Lumbar puncture	£268 (324.4)	Outpatient diagnostic procedure
	ICP bolt insertion	£509 (616.1)	Minimally invasive procedure
	Urgent intracranial procedure (revision of CSF shunt system)	£1893 (2291.4)	Including short-stay admission tariff, without critical care-level bed
Device	MScio telesensor	£2318 (2805.9)	Excludes cost of admission, implantation and shunt system and hand-held readers used with the device
	Sprung reservoir	£186 (225.1)	Excludes cost of admission, implantation and shunt system

CSF, cerebrospinal fluid; CT, computed tomography; GBP, Great Britian Pounds; ICP, intracranial pressure.

US dollar tariffs based on the foreign exchange rate of £1 = $1.2105.

#### Matching and Data Analysis

Matching was performed using a propensity scoring matching method,^12^ which aims to pair each telesensor subject with its closest counterpart using a k-nearest neighbors algorithm and propensity logit function based on the following criteria: age, sex, diagnostic category, type of shunt (ventriculo-peritoneal shunt [VPS] vs non-VPS), and whether the patient had a history of a shunt procedure (**Supplemental Digital Content 1**, http://links.lww.com/NEU/E143). All statistical analyses were performed in Python (v = 3.8.1), using pairwise *t*-tests or Wilcoxon signed rank tests depending on normality testing with 95% confidence intervals displayed where applicable.

We performed 3 further analyses. Owing to the imperfect nature of the matching process, we performed a sensitivity analysis which would assess 2-year financial differences, covarying the stringency of the matching process to assess if differences would be maintained. Second, because several model variables are likely to interact, we also performed a multivariate linear regression to identify the independent influence of reservoir type on total costs using the complete data set. For simplification, the control group cost was assumed to have a Sprung reservoir (Table 1). Finally, we attempted to find the break-even point at which savings began to be made.

Although the analyses in this study were exploratory, adjustments (using the Benjamini-Hochberg method) for multiple end points are shown where relevant, otherwise a *P* value of less than .05 was confirmed to be significant. Sample size was determined pragmatically based on the total number of telesensor patients available who met the study inclusion criteria in the allocated period. We performed post hoc power calculations based on an alpha of 0.05 and effect sizes based on differences in total number of encounters over 2 years between groups and found that both parametric and nonparametric independent group-wise analyses were sufficiently powered (β > 0.8) [G*Power, v = 3.1].

## RESULTS

### Patient Demographics and Preimplant Encounter History

A total of 136 patients met the inclusion criteria for the study (74 telesensor, 62 controls). After propensity matching, 48 pairs remained (Table 2). Matched controls were not statistically different from the telesensor group with respect to age, sex, primary diagnosis, and type of shunt (Table 2, **Supplemental Digital Content 1** [http://links.lww.com/NEU/E143]). When comparing encounters in the 2 years before shunt insertion, control patients were not significantly different with respect to inpatient attendances or invasive procedures or by number of secondary surgeries across the cohort (Table 2).

**TABLE 2.** Preimplantation Pairwise Comparison Between Matched Telesensor Patients and Standard Reservoir Controls With Number of Encounters Evaluated Over the Preceding 2-Year Period

	Telesensor	Control	*P*
n	48	48	—
Mean age in years (SD)	38.3 (14.7)	36.0 (14.1)	.30
Sex (F:M)	39:9	38:10	1.00
Diagnostic category	IIH = 16	IIH = 18	.99
	Congenital = 20	Congenital = 18	
	Tumor = 2	Tumor = 2	
	NPH = 2	NPH = 3	
	Other = 8^a^	Other = 7^a^	
Implantation			
Mean date	16/2/2017	8/4/2016	—
Primary vs revision	Primary = 26 Revision = 22	Primary = 28 Revision = 20	.84
VPS vs non-VPS	VPS = 37 non-VPS = 11	VPS = 35 non-VPS = 13	.81
Mean preimplantation encounters per patient (SD)			
Neurosurgical outpatient clinic	3.53 (3.00)	2.45 (2.80)	.07
Neurology outpatient clinic	1.58 (2.58)	1.75 (3.39)	.80
Ophthalmology outpatient clinic	0.33 (0.91)	0.46 (1.29)	.55
Emergency room attendance	0.57 (1.70)	0.22 (0.83)	.19
Neurosurgical admission	1.92 (1.65)	1.82 (1.58)	.73
CT head	2.14 (4.31)	2.94 (6.23)	.88
X-ray (single body part)	2.14 (4.31)	2.94 (6.23)	.45
MRI head	1.49 (1.63)	2.65 (3.03)	.02
Lumbar puncture	0.27 (0.70)	0.24 (0.62)	.75
Insertion of ICP monitor	0.77 (0.95)	0.98 (0.93)	.30
CSF shunt revision	0.88 (1.07)	0.59 (1.25)	.22

CSF, cerebrospinal fluid; CT, computed tomography; ICP, intracranial pressure; IIH, idiopathic intracranial hypotension; NPH, normal pressure hydrocephalus; VPS, ventriculo-peritoneal shunt.

a Other included CSF leak, secondary hydrocephalus due to subarachnoid hemorrhage, pseudomeningocele, and aqueductal stenosis.

However, controls did tend to have less neurosurgical outpatient encounters and significantly less MRI scans (with on average, 1 less scan per patient in the period), although this would not have met the threshold after multiple comparison corrections. The full description of the cohort is given in the supplemental data (**Supplemental Digital Content 1**, http://links.lww.com/NEU/E143). Total costs for 2 years before surgery were not significantly different between matched pairs.

### Service Demand

**FIGURE 2.**
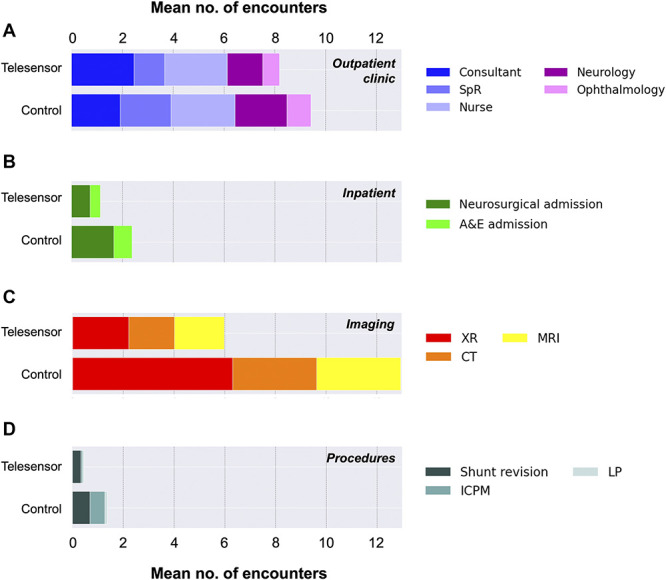
Differences and breakdown of hospital encounters for telesensor and control patients by **A**, outpatient attendances; **B**, inpatient admissions; **C**, imaging episodes; and **D**, invasive procedures. CT, computed tomography; ICPM, intracranial pressure monitoring; SpR, specialist registrar.

The usage of various elective and emergency facilities was evaluated over the 2-year period after implantation (Figure 2, Table 3).

**TABLE 3.** Comparison Between Patients With Telesensors Implanted as Vs Controls Who had a Standard Reservoir With Number of Postimplantation Encounters Evaluated Over the Proceeding 2-Year Period

Mean postimplantation encounters per patient (SD)	Telesensor	Control	Difference	*P*
Outpatient				
Neurosurgery				
All	5.72 (3.37)	6.38 (5.29)	−0.65	.48
Consultant	2.45 (3.15)	1.92 (3.96)	0.54	.46
SpR/resident	1.21 (1.35)	2.00 (2.10)	−1.08	.04
Nurse specialist	2.45 (2.09)	2.52 (3.48)	−0.06	.91
Nonneurosurgery				
Neurology	1.75 (2.50)	1.96 (2.89)	−0.21	.66
Ophthalmology	0.67 (1.41)	0.94 (1.39)	−0.27	.33
Inpatient				
Neurosurgery				
Admission	0.73 (1.14)	1.67 (2.64)	−0.94	.03
Bed days	7.48 (19.25)	13.1 (23.14)	−5.62	.22
A&E	0.40 (0.86)	0.71 (1.96)	−0.31	.36
Imaging				
X-ray	2.44 (2.82)	6.33 (7.97)	−3.90	.003^a^
CT	1.88 (3.12)	3.29 (3.71)	−1.42	.02
MRI	2.08 (3.24)	3.54 (5.02)	−1.46	.10
Procedures				
ICPM	0.04 (0.24)	0.52 (1.25)	−0.48	.01^a^
Lumbar puncture	0.04 (0.20)	0.08 (0.40)	−0.04	.53
Shunt revision	0.27 (0.61)	0.71 (1.64)	−0.44	.08

CT, computed tomography; ICPM, intracranial pressure monitoring; SpR, specialist registrar.

a Would remain significant after multiple comparison adjustment.

### Outpatient Attendance

On average, telesensor patients had 1.97 (SD 2.24) outpatient sensor checks in the 2 years after implantation and had 1.27 (SD 1.76) outpatient valve adjustments after implantation as compared with controls who had 1.58 (SD 2.07); however, this difference was not significant (difference = −0.31 [−1.13 to 0.50], statistic = −0.77, *P* = .44).

There was no significant difference in the total number of neurosurgery clinic appointments after implantation, although control patients were more likely to see a registrar (difference = 1.08 [0.04-1.54], statistic = 3.08, *P* = .04). There was no significant difference in neurology or ophthalmology attendances across matched pairs.

### Inpatient Admissions and Invasive Procedures

Control patients were more likely to require an unprompted neurosurgical admission after the shunt was inserted (difference = 0.94 [0.09-1.74], statistic = 2.23 *P* = .03) and were also more likely to require further ICP monitoring (difference = 0.48 [0.11-0.85], statistic = 2.57, *P* = .01). Control patients on average had a greater number of inpatient hospital days (13.1 days) as compared with telesensor patients (7.5 days), but this was not found to be significantly different on matched pair analysis. There was also no significant difference between frequency of further lumbar punctures or shunt revisions (although the latter was trending toward more in the control group).

### Imaging

Telesensor patients had significantly fewer imaging encounters across all modalities with, on average over 2 years, 3.9 less single body part x-rays (95% CI = 1.36-6.44, *P* = .003) and 1.4 less CT head scans (95% CI = 0.16-2.67, *P* = .02). Based on frequency of imaging encounters, over the 2-year period, telesensor patients received approximately, on average, 4.48 mSv (SD 6.64) of radiation, significantly less than control patients who received 8.48 mSv (SD 9.04, difference = 4.00 [1.02-6.98], *P* = .009), with this difference being equivalent to roughly 2 years of natural background radiation in the United Kingdom.^13^

### Costs

**FIGURE 3.**
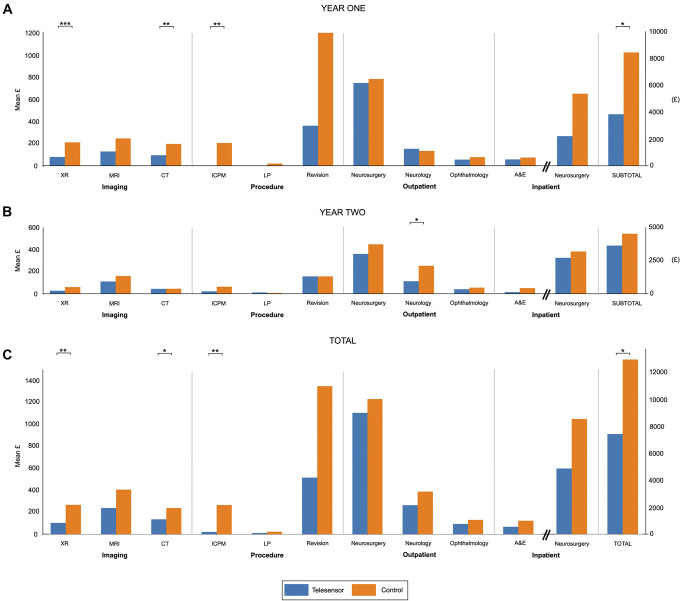
Mean pairwise cost differences between telesensor and control matched pairs at the end of **A**, year 1; **B**, year 2; **C**, and overall. *P < .05, **P < .01, ***P < .001. CT, computed tomography; ICPM, intracranial pressure monitoring.

The difference in costs between telesensor patients and controls during year 1, year 2, and overall are shown in Figure 3 and in the supplemental data (**Supplemental Digital Content 1**, http://links.lww.com/NEU/E143). Control patients accrued significantly greater costs related to intracranial pressure monitoring and CT and x-ray imaging in year 1 and trended toward greater costs for MRI and operative encounters. By the end of year 1, the mean pairwise saving (excluding the cost of the implant) was £4624 (95% CI = 102-9144, *P* = .03). In year 2 alone, control patients accrued greater costs across almost all the domains; however, these differences were not significant other than for neurology outpatient appointments (difference = £142 [7-276], *P* = .04). The mean saving in year 2 (excluding the cost of the implant) was £874 (95% CI = −2845 to 4593, *P* = .64). When accounting for both years, significant cost differences were found across the same domains as those found in year 1. The mean cumulative costs were £7391 among telesensor patients and £12 889 among control patients by the end of year 2 (difference = £5498 [6-10 989], *P* = .04). The main burden of cost for both groups was primarily related to neurosurgical inpatient admissions (Figure 3, **Supplemental Digital Content 1** [http://links.lww.com/NEU/E143]).

### Sensitivity, Multivariate, and Breakeven Analyses

We performed a sensitivity analysis to assess whether 2-year total costs would differ according to different levels of matching stringency (**Supplemental Digital Content 1**, http://links.lww.com/NEU/E143). Telesensor savings were maintained across stringency levels with a mean average of £5487 (+/− 1610) across matched pairs, although significant differences were found only up to 50 pairs.

Given that some model variables are likely to interact and patients were excluded during matching, a multivariate regression was performed for the entire data set (**Supplemental Digital Content 1**, http://links.lww.com/NEU/E143). After adjustment, we found that costs associated with telesensor use were significantly less with a saving of £5236 by the end of 2 years (95% CI = 171-10 300, t = −2.05, *P* = .04). Age, sex, and shunt type did not have significant associations in multivariate analyses nor did underlying diagnosis (**Supplemental Digital Content 1**, http://links.lww.com/NEU/E143).

**FIGURE 4.**
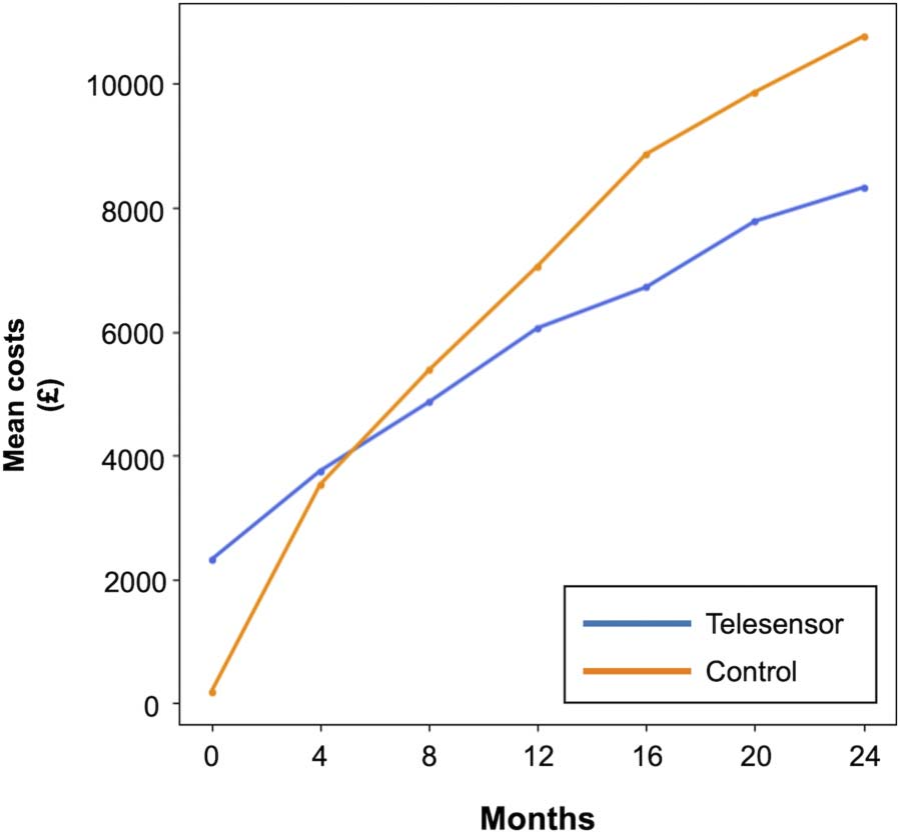
Two-year cumulative mean costs of telesensor and control patients after accounting for initial cost of the shunt reservoir with a break-even point identified at the crossover.

Both pairwise and multivariate analyses did not account for the initial financial burden of the cost of the reservoir. Using the full data set and including for the reservoir base cost, we attempted to identify the point at which a cost saving is made. As a mean average, the break-even point was found to be approximately less than 8 months after shunt insertion (Figure 4).

## DISCUSSION

### Summary of Results

This observational study assessed service demands and costs in adult hydrocephalus patients requiring CSF shunting who had an implanted telemetric sensor reservoir. After propensity matching, 48 telesensor patients were compared with 48 controls having nontelemetric reservoirs paired according to demographics, diagnosis, and shunt type. Using both univariate and multivariate analyses, on both matched and complete data sets, significant savings were found to be associated with use of telesensor reservoirs. After multivariate adjustment, the mean cumulative saving after 2 years was found to be £5236 ($6338) when using a telesensor reservoir (£5498 on matched pair analysis). Cost-savings were highly likely to be achieved within 8 months of clinical use after implantation. Finally of specific clinical importance was the significant reduction in imaging-associated radiation (4 mSv) over 2 years, for those with a telesensor.

### Interpretation and Context

Shunt insertion is a commonly performed adult neurosurgical procedure, with an estimated annual rate of 1500 patients undergoing primary shunt surgery in the United Kingdom.^14^ Within the first year, 15% of these patients require revision surgery, predominantly attributable to underdrainage and infection. In spite of the frequency of this surgery, the cost-effectiveness of shunt insertion and the various implantable devices used to assist and control CSF outflow are underrepresented within the existing literature, although collective evidence suggests the intervention is associated with significant improvements in Quality-Adjusted Life Years compared to nonsurgical patients and greater cost savings over time.^15-17^

The use of telemetric sensor reservoirs in both adult and pediatric CSF disorders has been increasing over the past decade. Although many studies have reported their early experiences and feasibility^18-21^ and application in clinical practice,^18,22,23^ very few have performed a financial-utility analysis—namely, whether the higher base cost of a more sophisticated reservoir translates to better clinical outcomes. In a study conducted by Bjornson et al, a cost-effectiveness analysis of Miethke telesensor reservoirs was performed on a cohort of 12 patients, including 3 children and 9 adults. Here, the expenses associated with patient investigations and interventions were analyzed during a 2-year period before and after telesensor insertion. After the telesensor insertion, a significant cost reduction of £6952 per patient was observed over a 2-year duration, along with a corresponding reduction in the frequency of investigations such as CT head scans, x-rays, and ICP monitoring. A larger cost reduction was found as compared with the more modest cost savings found in our work, may at least partially be due to their small heterogeneous sample, which included children, differences in institutional tariffs, and that costs were compared with the preimplantation 2 years rather than a matched control group.

### Limitations

We acknowledge several limitations in this exploratory study. First, this was a nonrandomized observational single-center study with a follow-up period of only 2 years. Both the matched pair and full cohort had a predisposition toward idiopathic intracranial hypotension and congenital hydrocephalus diagnoses. These diagnostic groups tend to be high service users as compared with those with acquired hydrocephalus. Although a like-for-like comparison was performed during matching and diagnosis was regressed as a covariate in multivariate tests, this issue limits the generalizability of the findings to other centers with more heterogeneous patient cohorts and for longer periods. In addition, although there was clinical equipoise in patient selection and patient-related variables were statistically controlled, one cannot completely mitigate for a selection bias with this study design. Second, there was no formal evaluation of “effectiveness” or quality of life. Third, other costs associated with labor and investigations or attendances outside the hospital center were not collected which may have influenced the results. Finally, further independent work is needed to assess the technical discrepancy between Miethke telesensor reservoirs readings and actual pressure and whether this becomes more apparent over time: “sensor drift,” as studied with the Raumedic Neurovent-P-tel.^24^

In spite of these issues, we highlight that our study is the first to formally and comprehensively assess the associated costs and service demands of shunt reservoir use in a large, realistic cohort of both chronic and subacute hydrocephalus patients. We applied a robust statistical design that uses both propensity matching andmultivariate techniques with results that are remarkably consistent and coherent.

## CONCLUSION

From an institutional perspective, the implantation of telesensors contributes to a reduction in service demand and a likely net financial saving. From a patient perspective, fewer appointments, invasive procedures, and less radiation exposure suggest an improvement in patient experience and safety. However, additional studies are needed to confirm the hypothesis that telesensors are cost-effective in the long-term. Nevertheless, the exploratory findings of this study highlight the potential benefits of telesensors in reducing the financial burden of neurosurgical departments and their potential to improve the overall management of hydrocephalus.

## Supplementary Material

SUPPLEMENTARY MATERIAL

## SUPPLEMENTAL DIGITAL CONTENT

**Supplemental Digital Content 1. Methods, Results.** The Supplemental Digital Content expands on the Methods to further explain the matching process and Results for additional specific analyses. **Figure 1.** Sensitivity analysis. **Table 1.** comparison of telesensor patients and controls with respect to specific diagnoses. **Table 2.** demographic details of the full data set. **Table 3.** Mean annual and total pairwise cost differences. **Table 4.** Multivariate linear regression.

## References

[1] Stagno V Navarrete EA Mirone G Esposito F. Management of hydrocephalus around the World. World Neurosurg. 2013;79(2 Suppl):S23.e17-S23.e20. 10.1016/j.wneu.2012.02.004 22381848

[2] Yamashita N Kamiya K Yamada K. Experience with a programmable valve shunt system. J Neurosurg. 1999;91(1):26-31. 10389876 10.3171/jns.1999.91.1.0026

[3] Khawari S Pandit A Watkins L Toma A Thorne L. Can clinicians correctly predict intracranial pressure state based on clinical symptoms alone in shunted patients? J Neurosurg Sci. Published online November 23, 2023. doi: 10.23736/S0390-5616.23.06065-4. 10.23736/S0390-5616.23.06065-4 37997322

[4] Friedman DI Quiros PA Subramanian PS Headache in idiopathic intracranial hypertension: findings from the idiopathic intracranial hypertension treatment trial. Headache. 2017;57(8):1195-1205. 28752894 10.1111/head.13153 PMC5799151

[5] Antes S Tschan CA Heckelmann M Breuskin D Oertel J. Telemetric intracranial pressure monitoring with the raumedic neurovent P-tel. World Neurosurg. 2016;91:133-148. 27060515 10.1016/j.wneu.2016.03.096

[6] Antes S Stadie A Müller S Linsler S Breuskin D Oertel J. Intracranial pressure-guided shunt valve adjustments with the miethke sensor reservoir. World Neurosurg. 2018;109:e642-e650. 29054776 10.1016/j.wneu.2017.10.044

[7] Freimann FB Schulz M Haberl H Thomale UW. Feasibility of telemetric ICP-guided valve adjustments for complex shunt therapy. Childs Nerv Syst. 2014;30(4):689-697. 24264382 10.1007/s00381-013-2324-0

[8] Adam A Robison J Lu J Abstracts from hydrocephalus 2016. Fluids Barriers CNS. 2017;14(Suppl 1):15. 28929972 10.1186/s12987-017-0054-5 PMC5471936

[9] Shellock FG Knebel J Prat AD. Evaluation of MRI issues for a new neurological implant, the Sensor Reservoir. Magn Reson Imaging. 2013;31(7):1245-1250. 23602731 10.1016/j.mri.2013.03.012

[10] Ertl P Hermann E Heissler H Krauss J. Telemetric intracranial pressure recording via a shunt system integrated sensor: a safety and feasibility study. J Neurol Surg A, Cent Eur Neurosurg. 2017;78(6):572-575. 28586937 10.1055/s-0037-1603632

[11] Siegel JE Weinstein MC Russell LB Gold MR. Recommendations for reporting cost-effectiveness analyses. Panel on cost-effectiveness in health and medicine. JAMA. 1996;276(16):1339-1341. 8861994 10.1001/jama.276.16.1339

[12] Kline A Luo Y. PsmPy: a package for retrospective cohort matching in python. Annu Int Conf IEEE Eng Med Biol Soc IEEE Eng Med Biol Soc Annu Int Conf. 2022;2022:1354-1357. 10.1109/EMBC48229.2022.9871333 36086543

[13] Ionising Radiation: Dose Comparisons. Accessed July 9, 2023. https://www.gov.uk/government/publications/ionising-radiation-dose-comparisons/ionising-radiation-dose-comparisons

[14] Fernández-Méndez R Richards HK Seeley HM Current epidemiology of cerebrospinal fluid shunt surgery in the UK and Ireland (2004-2013). J Neurol Neurosurg Psychiatry. 2019;90(7):747-754. 30910858 10.1136/jnnp-2018-319927 PMC6585267

[15] Kameda M Yamada S Atsuchi M Cost-effectiveness analysis of shunt surgery for idiopathic normal pressure hydrocephalus based on the SINPHONI and SINPHONI-2 trials. Acta Neurochir. 2017;159(6):995-1003. 28251346 10.1007/s00701-017-3115-2

[16] Tullberg M Persson J Petersen J Hellström P Wikkelsø C Lundgren-Nilsson Å. Shunt surgery in idiopathic normal pressure hydrocephalus is cost-effective—a cost utility analysis. Acta Neurochir. 2018;160(3):509-518. 29150794 10.1007/s00701-017-3394-7 PMC5807454

[17] Mallucci CL Jenkinson MD Conroy EJ Antibiotic or silver versus standard ventriculoperitoneal shunts (BASICS): a multicentre, single-blinded, randomised trial and economic evaluation. Lancet. 2019;394(10208):1530-1539. 31522843 10.1016/S0140-6736(19)31603-4 PMC6999649

[18] Bjornson A Henderson D Lawrence E McMullan J Ushewokunze S. The sensor reservoir—does it change management? Acta Neurochir. 2021;163(4):1087-1095. 33587185 10.1007/s00701-021-04729-y

[19] Norager NH Lilja-Cyron A Hansen TS Juhler M. Deciding on appropriate telemetric intracranial pressure monitoring system. World Neurosurg. 2019;126:564-569. 30898734 10.1016/j.wneu.2019.03.077

[20] Müller SJ Freimann FB von der Brelie C Rohde V Schatlo B. Test-retest reliability of outpatient telemetric intracranial pressure measurements in shunt-dependent patients with hydrocephalus and idiopathic intracranial hypertension. World Neurosurg. 2019;131:e74-e80. 31295619 10.1016/j.wneu.2019.07.014

[21] Pennacchietti V Prinz V Schaumann A Finger T Schulz M Thomale UW. Single center experiences with telemetric intracranial pressure measurements in patients with CSF circulation disturbances. Acta Neurochir. 2020;162(10):2487-2497. 32495080 10.1007/s00701-020-04421-7 PMC7496065

[22] Richard KE Block FR Weiser RR. First clinical results with a telemetric shunt-integrated ICP-sensor. Neurol Res. 1999;21(1):117-120. 10048069 10.1080/01616412.1999.11740906

[23] Pedersen SH Norager NH Lilja-Cyron A Juhler M. Telemetric intracranial pressure monitoring in children. Childs Nerv Syst. 2020;36(1):49-58. 31309286 10.1007/s00381-019-04271-4

[24] Norager NH Lilja-Cyron A Bjarkam CR Duus S Juhler M. Telemetry in intracranial pressure monitoring: sensor survival and drift. Acta Neurochir. 2018;160(11):2137-2144. 30267207 10.1007/s00701-018-3691-9

